# Supplementary material for: HIVprotI: an integrated web based platform for prediction and design of HIV proteins inhibitors
Source: J Cheminform. 2018 Mar 9;10:12. doi: 10.1186/s13321-018-0266-y (PMC5845081; doi:10.1186/s13321-018-0266-y)
Supplement: Supplementary file 2 — Additional file 2. Source code of HIVProtI web server. [file 13321_2018_266_MOESM2_ESM.zip › HIVprotI_Source-code/hivprotI/weka-3-6-6/doc/weka/filters/unsupervised/attribute/StringToWordVector.html]

StringToWordVector


---


|  |  |  |
| --- | --- | --- |
| **Overview** \| **Package** \| **Class** \| **Tree** \| **Deprecated** \| **Index** \| **Help** \| **Weka's home** | |  |
| **PREV CLASS**   **NEXT CLASS** | **FRAMES**    **NO FRAMES**     **All Classes** |  |
| SUMMARY: NESTED \| FIELD \| CONSTR \| METHOD | DETAIL: FIELD \| CONSTR \| METHOD |  |


---


## weka.filters.unsupervised.attribute Class StringToWordVector

```
java.lang.Object
  weka.filters.Filter
      weka.filters.unsupervised.attribute.StringToWordVector
```

**All Implemented Interfaces:**: java.io.Serializable, CapabilitiesHandler, OptionHandler, RevisionHandler, UnsupervisedFilter

---

``` public class StringToWordVector extends Filter implements UnsupervisedFilter, OptionHandler ```

Converts String attributes into a set of attributes representing word occurrence (depending on the tokenizer) information from the text contained in the strings. The set of words (attributes) is determined by the first batch filtered (typically training data).


Valid options are:

```
 -C
  Output word counts rather than boolean word presence.
```

```
 -R <index1,index2-index4,...>
  Specify list of string attributes to convert to words (as weka Range).
  (default: select all string attributes)
```

```
 -V
  Invert matching sense of column indexes.
```

```
 -P <attribute name prefix>
  Specify a prefix for the created attribute names.
  (default: "")
```

```
 -W <number of words to keep>
  Specify approximate number of word fields to create.
  Surplus words will be discarded..
  (default: 1000)
```

```
 -prune-rate <rate as a percentage of dataset>
  Specify the rate (e.g., every 10% of the input dataset) at which to periodically prune the dictionary.
  -W prunes after creating a full dictionary. You may not have enough memory for this approach.
  (default: no periodic pruning)
```

```
 -T
  Transform the word frequencies into log(1+fij)
  where fij is the frequency of word i in jth document(instance).
```

```
 -I
  Transform each word frequency into:
  fij*log(num of Documents/num of documents containing word i)
    where fij if frequency of word i in jth document(instance)
```

```
 -N
  Whether to 0=not normalize/1=normalize all data/2=normalize test data only
  to average length of training documents (default 0=don't normalize).
```

```
 -L
  Convert all tokens to lowercase before adding to the dictionary.
```

```
 -S
  Ignore words that are in the stoplist.
```

```
 -stemmer <spec>
  The stemmering algorihtm (classname plus parameters) to use.
```

```
 -M <int>
  The minimum term frequency (default = 1).
```

```
 -O
  If this is set, the maximum number of words and the 
  minimum term frequency is not enforced on a per-class 
  basis but based on the documents in all the classes 
  (even if a class attribute is set).
```

```
 -stopwords <file>
  A file containing stopwords to override the default ones.
  Using this option automatically sets the flag ('-S') to use the
  stoplist if the file exists.
  Format: one stopword per line, lines starting with '#'
  are interpreted as comments and ignored.
```

```
 -tokenizer <spec>
  The tokenizing algorihtm (classname plus parameters) to use.
  (default: weka.core.tokenizers.WordTokenizer)
```

**Version:**
:   $Revision: 5547 $

**Author:**
:   Len Trigg (len@reeltwo.com), Stuart Inglis (stuart@reeltwo.com), Gordon Paynter (gordon.paynter@ucr.edu), Asrhaf M. Kibriya (amk14@cs.waikato.ac.nz)

**See Also:**: `Stopwords`, Serialized Form

---

| **Field Summary** | |
| --- | --- |
| `static int` | `FILTER_NONE`             normalization: No normalization. |
| `static int` | `FILTER_NORMALIZE_ALL`             normalization: Normalize all data. |
| `static int` | `FILTER_NORMALIZE_TEST_ONLY`             normalization: Normalize test data only. |
| `static Tag[]` | `TAGS_FILTER`             Specifies whether document's (instance's) word frequencies are to be normalized. |


**Constructor Summary**

`StringToWordVector()`
  
          Default constructor.

`StringToWordVector(int wordsToKeep)`
  
          Constructor that allows specification of the target number of words
in the output.


| **Method Summary** | |
| --- | --- |
| `java.lang.String` | `attributeIndicesTipText()`             Returns the tip text for this property. |
| `java.lang.String` | `attributeNamePrefixTipText()`             Returns the tip text for this property. |
| `boolean` | `batchFinished()`             Signify that this batch of input to the filter is finished. |
| `java.lang.String` | `doNotOperateOnPerClassBasisTipText()`             Returns the tip text for this property. |
| `java.lang.String` | `getAttributeIndices()`             Gets the current range selection. |
| `java.lang.String` | `getAttributeNamePrefix()`             Get the attribute name prefix. |
| `Capabilities` | `getCapabilities()`             Returns the Capabilities of this filter. |
| `boolean` | `getDoNotOperateOnPerClassBasis()`             Get the DoNotOperateOnPerClassBasis value. |
| `boolean` | `getIDFTransform()`             Sets whether if the word frequencies in a document should be transformed into:   fij\*log(num of Docs/num of Docs with word i)   where fij is the frequency of word i in document(instance) j. |
| `boolean` | `getInvertSelection()`             Gets whether the supplied columns are to be processed or skipped. |
| `boolean` | `getLowerCaseTokens()`             Gets whether if the tokens are to be downcased or not. |
| `int` | `getMinTermFreq()`             Get the MinTermFreq value. |
| `SelectedTag` | `getNormalizeDocLength()`             Gets whether if the word frequencies for a document (instance) should be normalized or not. |
| `java.lang.String[]` | `getOptions()`             Gets the current settings of the filter. |
| `boolean` | `getOutputWordCounts()`             Gets whether output instances contain 0 or 1 indicating word presence, or word counts. |
| `double` | `getPeriodicPruning()`             Gets the rate at which the dictionary is periodically pruned, as a percentage of the dataset size. |
| `java.lang.String` | `getRevision()`             Returns the revision string. |
| `Range` | `getSelectedRange()`             Get the value of m\_SelectedRange. |
| `Stemmer` | `getStemmer()`             Returns the current stemming algorithm, null if none is used. |
| `java.io.File` | `getStopwords()`             returns the file used for obtaining the stopwords, if the file represents a directory then the default ones are used. |
| `boolean` | `getTFTransform()`             Gets whether if the word frequencies should be transformed into log(1+fij) where fij is the frequency of word i in document(instance) j. |
| `Tokenizer` | `getTokenizer()`             Returns the current tokenizer algorithm. |
| `boolean` | `getUseStoplist()`             Gets whether if the words on the stoplist are to be ignored (The stoplist is in weka.core.StopWords). |
| `int` | `getWordsToKeep()`             Gets the number of words (per class if there is a class attribute assigned) to attempt to keep. |
| `java.lang.String` | `globalInfo()`             Returns a string describing this filter. |
| `java.lang.String` | `IDFTransformTipText()`             Returns the tip text for this property. |
| `boolean` | `input(Instance instance)`             Input an instance for filtering. |
| `java.lang.String` | `invertSelectionTipText()`             Returns the tip text for this property. |
| `java.util.Enumeration` | `listOptions()`             Returns an enumeration describing the available options. |
| `java.lang.String` | `lowerCaseTokensTipText()`             Returns the tip text for this property. |
| `static void` | `main(java.lang.String[] argv)`             Main method for testing this class. |
| `java.lang.String` | `minTermFreqTipText()`             Returns the tip text for this property. |
| `java.lang.String` | `normalizeDocLengthTipText()`             Returns the tip text for this property. |
| `java.lang.String` | `outputWordCountsTipText()`             Returns the tip text for this property. |
| `java.lang.String` | `periodicPruningTipText()`             Returns the tip text for this property. |
| `void` | `setAttributeIndices(java.lang.String rangeList)`             Sets which attributes are to be worked on. |
| `void` | `setAttributeIndicesArray(int[] attributes)`             Sets which attributes are to be processed. |
| `void` | `setAttributeNamePrefix(java.lang.String newPrefix)`             Set the attribute name prefix. |
| `void` | `setDoNotOperateOnPerClassBasis(boolean newDoNotOperateOnPerClassBasis)`             Set the DoNotOperateOnPerClassBasis value. |
| `void` | `setIDFTransform(boolean IDFTransform)`             Sets whether if the word frequencies in a document should be transformed into:   fij\*log(num of Docs/num of Docs with word i)   where fij is the frequency of word i in document(instance) j. |
| `boolean` | `setInputFormat(Instances instanceInfo)`             Sets the format of the input instances. |
| `void` | `setInvertSelection(boolean invert)`             Sets whether selected columns should be processed or skipped. |
| `void` | `setLowerCaseTokens(boolean downCaseTokens)`             Sets whether if the tokens are to be downcased or not. |
| `void` | `setMinTermFreq(int newMinTermFreq)`             Set the MinTermFreq value. |
| `void` | `setNormalizeDocLength(SelectedTag newType)`             Sets whether if the word frequencies for a document (instance) should be normalized or not. |
| `void` | `setOptions(java.lang.String[] options)`             Parses a given list of options. |
| `void` | `setOutputWordCounts(boolean outputWordCounts)`             Sets whether output instances contain 0 or 1 indicating word presence, or word counts. |
| `void` | `setPeriodicPruning(double newPeriodicPruning)`             Sets the rate at which the dictionary is periodically pruned, as a percentage of the dataset size. |
| `void` | `setSelectedRange(java.lang.String newSelectedRange)`             Set the value of m\_SelectedRange. |
| `void` | `setStemmer(Stemmer value)`             the stemming algorithm to use, null means no stemming at all (i.e., the NullStemmer is used). |
| `void` | `setStopwords(java.io.File value)`             sets the file containing the stopwords, null or a directory unset the stopwords. |
| `void` | `setTFTransform(boolean TFTransform)`             Sets whether if the word frequencies should be transformed into log(1+fij) where fij is the frequency of word i in document(instance) j. |
| `void` | `setTokenizer(Tokenizer value)`             the tokenizer algorithm to use. |
| `void` | `setUseStoplist(boolean useStoplist)`             Sets whether if the words that are on a stoplist are to be ignored (The stop list is in weka.core.StopWords). |
| `void` | `setWordsToKeep(int newWordsToKeep)`             Sets the number of words (per class if there is a class attribute assigned) to attempt to keep. |
| `java.lang.String` | `stemmerTipText()`             Returns the tip text for this property. |
| `java.lang.String` | `stopwordsTipText()`             Returns the tip text for this property. |
| `java.lang.String` | `TFTransformTipText()`             Returns the tip text for this property. |
| `java.lang.String` | `tokenizerTipText()`             Returns the tip text for this property. |
| `java.lang.String` | `useStoplistTipText()`             Returns the tip text for this property. |
| `java.lang.String` | `wordsToKeepTipText()`             Returns the tip text for this property. |

**Methods inherited from class weka.filters.Filter**

`batchFilterFile, filterFile, getCapabilities, getOutputFormat, isFirstBatchDone, isNewBatch, isOutputFormatDefined, makeCopies, makeCopy, numPendingOutput, output, outputPeek, toString, useFilter, wekaStaticWrapper`

**Methods inherited from class java.lang.Object**

`equals, getClass, hashCode, notify, notifyAll, wait, wait, wait`

**Field Detail**

### FILTER\_NONE

```
public static final int FILTER_NONE
```

:   normalization: No normalization.

    **See Also:**: Constant Field Values

---


### FILTER\_NORMALIZE\_ALL

```
public static final int FILTER_NORMALIZE_ALL
```

:   normalization: Normalize all data.

    **See Also:**: Constant Field Values

---


### FILTER\_NORMALIZE\_TEST\_ONLY

```
public static final int FILTER_NORMALIZE_TEST_ONLY
```

:   normalization: Normalize test data only.

    **See Also:**: Constant Field Values

---


### TAGS\_FILTER

```
public static final Tag[] TAGS_FILTER
```

:   Specifies whether document's (instance's) word frequencies are
    to be normalized. The are normalized to average length of
    documents specified as input format.


**Constructor Detail**

### StringToWordVector

```
public StringToWordVector()
```

:   Default constructor. Targets 1000 words in the output.

---


### StringToWordVector

```
public StringToWordVector(int wordsToKeep)
```

:   Constructor that allows specification of the target number of words
    in the output.

    **Parameters:**: `wordsToKeep` - the number of words in the output vector (per class if assigned).


**Method Detail**

### listOptions

```
public java.util.Enumeration listOptions()
```

:   Returns an enumeration describing the available options.

    :   **Specified by:**: `listOptions` in interface `OptionHandler`
    :   **Returns:**: an enumeration of all the available options

---


### setOptions

```
public void setOptions(java.lang.String[] options)
                throws java.lang.Exception
```

:   Parses a given list of options.


    Valid options are:

    ```
     -C
      Output word counts rather than boolean word presence.
    ```

    ```
     -R <index1,index2-index4,...>
      Specify list of string attributes to convert to words (as weka Range).
      (default: select all string attributes)
    ```

    ```
     -V
      Invert matching sense of column indexes.
    ```

    ```
     -P <attribute name prefix>
      Specify a prefix for the created attribute names.
      (default: "")
    ```

    ```
     -W <number of words to keep>
      Specify approximate number of word fields to create.
      Surplus words will be discarded..
      (default: 1000)
    ```

    ```
     -prune-rate <rate as a percentage of dataset>
      Specify the rate (e.g., every 10% of the input dataset) at which to periodically prune the dictionary.
      -W prunes after creating a full dictionary. You may not have enough memory for this approach.
      (default: no periodic pruning)
    ```

    ```
     -T
      Transform the word frequencies into log(1+fij)
      where fij is the frequency of word i in jth document(instance).
    ```

    ```
     -I
      Transform each word frequency into:
      fij*log(num of Documents/num of documents containing word i)
        where fij if frequency of word i in jth document(instance)
    ```

    ```
     -N
      Whether to 0=not normalize/1=normalize all data/2=normalize test data only
      to average length of training documents (default 0=don't normalize).
    ```

    ```
     -L
      Convert all tokens to lowercase before adding to the dictionary.
    ```

    ```
     -S
      Ignore words that are in the stoplist.
    ```

    ```
     -stemmer <spec>
      The stemmering algorihtm (classname plus parameters) to use.
    ```

    ```
     -M <int>
      The minimum term frequency (default = 1).
    ```

    ```
     -O
      If this is set, the maximum number of words and the 
      minimum term frequency is not enforced on a per-class 
      basis but based on the documents in all the classes 
      (even if a class attribute is set).
    ```

    ```
     -stopwords <file>
      A file containing stopwords to override the default ones.
      Using this option automatically sets the flag ('-S') to use the
      stoplist if the file exists.
      Format: one stopword per line, lines starting with '#'
      are interpreted as comments and ignored.
    ```

    ```
     -tokenizer <spec>
      The tokenizing algorihtm (classname plus parameters) to use.
      (default: weka.core.tokenizers.WordTokenizer)
    ```

    :   **Specified by:**: `setOptions` in interface `OptionHandler`
    :   **Parameters:**: `options` - the list of options as an array of strings **Throws:**: `java.lang.Exception` - if an option is not supported

---


### getOptions

```
public java.lang.String[] getOptions()
```

:   Gets the current settings of the filter.

    :   **Specified by:**: `getOptions` in interface `OptionHandler`
    :   **Returns:**: an array of strings suitable for passing to setOptions

---


### getCapabilities

```
public Capabilities getCapabilities()
```

:   Returns the Capabilities of this filter.

    :   **Specified by:**: `getCapabilities` in interface `CapabilitiesHandler` **Overrides:**: `getCapabilities` in class `Filter`
    :   **Returns:**: the capabilities of this object **See Also:**: `Capabilities`

---


### setInputFormat

```
public boolean setInputFormat(Instances instanceInfo)
                       throws java.lang.Exception
```

:   Sets the format of the input instances.

    :   **Overrides:**: `setInputFormat` in class `Filter`
    :   **Parameters:**: `instanceInfo` - an Instances object containing the input instance structure (any instances contained in the object are ignored - only the structure is required). **Returns:**: true if the outputFormat may be collected immediately **Throws:**: `java.lang.Exception` - if the input format can't be set successfully

---


### input

```
public boolean input(Instance instance)
              throws java.lang.Exception
```

:   Input an instance for filtering. Filter requires all
    training instances be read before producing output.

    :   **Overrides:**: `input` in class `Filter`
    :   **Parameters:**: `instance` - the input instance. **Returns:**: true if the filtered instance may now be collected with output(). **Throws:**: `java.lang.IllegalStateException` - if no input structure has been defined.: `java.lang.NullPointerException` - if the input format has not been defined.: `java.lang.Exception` - if the input instance was not of the correct format or if there was a problem with the filtering.

---


### batchFinished

```
public boolean batchFinished()
                      throws java.lang.Exception
```

:   Signify that this batch of input to the filter is finished.
    If the filter requires all instances prior to filtering,
    output() may now be called to retrieve the filtered instances.

    :   **Overrides:**: `batchFinished` in class `Filter`
    :   **Returns:**: true if there are instances pending output. **Throws:**: `java.lang.IllegalStateException` - if no input structure has been defined.: `java.lang.NullPointerException` - if no input structure has been defined,: `java.lang.Exception` - if there was a problem finishing the batch.

---


### globalInfo

```
public java.lang.String globalInfo()
```

:   Returns a string describing this filter.

    :   **Returns:**: a description of the filter suitable for displaying in the explorer/experimenter gui

---


### getOutputWordCounts

```
public boolean getOutputWordCounts()
```

:   Gets whether output instances contain 0 or 1 indicating word
    presence, or word counts.

    :   **Returns:**: true if word counts should be output.

---


### setOutputWordCounts

```
public void setOutputWordCounts(boolean outputWordCounts)
```

:   Sets whether output instances contain 0 or 1 indicating word
    presence, or word counts.

    :   **Parameters:**: `outputWordCounts` - true if word counts should be output.

---


### outputWordCountsTipText

```
public java.lang.String outputWordCountsTipText()
```

:   Returns the tip text for this property.

    :   **Returns:**: tip text for this property suitable for displaying in the explorer/experimenter gui

---


### getSelectedRange

```
public Range getSelectedRange()
```

:   Get the value of m\_SelectedRange.

    :   **Returns:**: Value of m\_SelectedRange.

---


### setSelectedRange

```
public void setSelectedRange(java.lang.String newSelectedRange)
```

:   Set the value of m\_SelectedRange.

    :   **Parameters:**: `newSelectedRange` - Value to assign to m\_SelectedRange.

---


### attributeIndicesTipText

```
public java.lang.String attributeIndicesTipText()
```

:   Returns the tip text for this property.

    :   **Returns:**: tip text for this property suitable for displaying in the explorer/experimenter gui

---


### getAttributeIndices

```
public java.lang.String getAttributeIndices()
```

:   Gets the current range selection.

    :   **Returns:**: a string containing a comma separated list of ranges

---


### setAttributeIndices

```
public void setAttributeIndices(java.lang.String rangeList)
```

:   Sets which attributes are to be worked on.

    :   **Parameters:**: `rangeList` - a string representing the list of attributes. Since the string will typically come from a user, attributes are indexed from 1. eg: first-3,5,6-last **Throws:**: `java.lang.IllegalArgumentException` - if an invalid range list is supplied

---


### setAttributeIndicesArray

```
public void setAttributeIndicesArray(int[] attributes)
```

:   Sets which attributes are to be processed.

    :   **Parameters:**: `attributes` - an array containing indexes of attributes to process. Since the array will typically come from a program, attributes are indexed from 0. **Throws:**: `java.lang.IllegalArgumentException` - if an invalid set of ranges is supplied

---


### invertSelectionTipText

```
public java.lang.String invertSelectionTipText()
```

:   Returns the tip text for this property.

    :   **Returns:**: tip text for this property suitable for displaying in the explorer/experimenter gui

---


### getInvertSelection

```
public boolean getInvertSelection()
```

:   Gets whether the supplied columns are to be processed or skipped.

    :   **Returns:**: true if the supplied columns will be kept

---


### setInvertSelection

```
public void setInvertSelection(boolean invert)
```

:   Sets whether selected columns should be processed or skipped.

    :   **Parameters:**: `invert` - the new invert setting

---


### getAttributeNamePrefix

```
public java.lang.String getAttributeNamePrefix()
```

:   Get the attribute name prefix.

    :   **Returns:**: The current attribute name prefix.

---


### setAttributeNamePrefix

```
public void setAttributeNamePrefix(java.lang.String newPrefix)
```

:   Set the attribute name prefix.

    :   **Parameters:**: `newPrefix` - String to use as the attribute name prefix.

---


### attributeNamePrefixTipText

```
public java.lang.String attributeNamePrefixTipText()
```

:   Returns the tip text for this property.

    :   **Returns:**: tip text for this property suitable for displaying in the explorer/experimenter gui

---


### getWordsToKeep

```
public int getWordsToKeep()
```

:   Gets the number of words (per class if there is a class attribute
    assigned) to attempt to keep.

    :   **Returns:**: the target number of words in the output vector (per class if assigned).

---


### setWordsToKeep

```
public void setWordsToKeep(int newWordsToKeep)
```

:   Sets the number of words (per class if there is a class attribute
    assigned) to attempt to keep.

    :   **Parameters:**: `newWordsToKeep` - the target number of words in the output vector (per class if assigned).

---


### wordsToKeepTipText

```
public java.lang.String wordsToKeepTipText()
```

:   Returns the tip text for this property.

    :   **Returns:**: tip text for this property suitable for displaying in the explorer/experimenter gui

---


### getPeriodicPruning

```
public double getPeriodicPruning()
```

:   Gets the rate at which the dictionary is periodically pruned, as a
    percentage of the dataset size.

    :   **Returns:**: the rate at which the dictionary is periodically pruned

---


### setPeriodicPruning

```
public void setPeriodicPruning(double newPeriodicPruning)
```

:   Sets the rate at which the dictionary is periodically pruned, as a
    percentage of the dataset size.

    :   **Parameters:**: `newPeriodicPruning` - the rate at which the dictionary is periodically pruned

---


### periodicPruningTipText

```
public java.lang.String periodicPruningTipText()
```

:   Returns the tip text for this property.

    :   **Returns:**: tip text for this property suitable for displaying in the explorer/experimenter gui

---


### getTFTransform

```
public boolean getTFTransform()
```

:   Gets whether if the word frequencies should be transformed into
    log(1+fij) where fij is the frequency of word i in document(instance) j.

    :   **Returns:**: true if word frequencies are to be transformed.

---


### setTFTransform

```
public void setTFTransform(boolean TFTransform)
```

:   Sets whether if the word frequencies should be transformed into
    log(1+fij) where fij is the frequency of word i in document(instance) j.

    :   **Parameters:**: `TFTransform` - true if word frequencies are to be transformed.

---


### TFTransformTipText

```
public java.lang.String TFTransformTipText()
```

:   Returns the tip text for this property.

    :   **Returns:**: tip text for this property suitable for displaying in the explorer/experimenter gui

---


### getIDFTransform

```
public boolean getIDFTransform()
```

:   Sets whether if the word frequencies in a document should be transformed
    into:   
    fij\*log(num of Docs/num of Docs with word i)   
    where fij is the frequency of word i in document(instance) j.

    :   **Returns:**: true if the word frequencies are to be transformed.

---


### setIDFTransform

```
public void setIDFTransform(boolean IDFTransform)
```

:   Sets whether if the word frequencies in a document should be transformed
    into:   
    fij\*log(num of Docs/num of Docs with word i)   
    where fij is the frequency of word i in document(instance) j.

    :   **Parameters:**: `IDFTransform` - true if the word frequecies are to be transformed

---


### IDFTransformTipText

```
public java.lang.String IDFTransformTipText()
```

:   Returns the tip text for this property.

    :   **Returns:**: tip text for this property suitable for displaying in the explorer/experimenter gui

---


### getNormalizeDocLength

```
public SelectedTag getNormalizeDocLength()
```

:   Gets whether if the word frequencies for a document (instance) should
    be normalized or not.

    :   **Returns:**: true if word frequencies are to be normalized.

---


### setNormalizeDocLength

```
public void setNormalizeDocLength(SelectedTag newType)
```

:   Sets whether if the word frequencies for a document (instance) should
    be normalized or not.

    :   **Parameters:**: `newType` - the new type.

---


### normalizeDocLengthTipText

```
public java.lang.String normalizeDocLengthTipText()
```

:   Returns the tip text for this property.

    :   **Returns:**: tip text for this property suitable for displaying in the explorer/experimenter gui

---


### getLowerCaseTokens

```
public boolean getLowerCaseTokens()
```

:   Gets whether if the tokens are to be downcased or not.

    :   **Returns:**: true if the tokens are to be downcased.

---


### setLowerCaseTokens

```
public void setLowerCaseTokens(boolean downCaseTokens)
```

:   Sets whether if the tokens are to be downcased or not. (Doesn't affect
    non-alphabetic characters in tokens).

    :   **Parameters:**: `downCaseTokens` - should be true if only lower case tokens are to be formed.

---


### doNotOperateOnPerClassBasisTipText

```
public java.lang.String doNotOperateOnPerClassBasisTipText()
```

:   Returns the tip text for this property.

    :   **Returns:**: tip text for this property suitable for displaying in the explorer/experimenter gui

---


### getDoNotOperateOnPerClassBasis

```
public boolean getDoNotOperateOnPerClassBasis()
```

:   Get the DoNotOperateOnPerClassBasis value.

    :   **Returns:**: the DoNotOperateOnPerClassBasis value.

---


### setDoNotOperateOnPerClassBasis

```
public void setDoNotOperateOnPerClassBasis(boolean newDoNotOperateOnPerClassBasis)
```

:   Set the DoNotOperateOnPerClassBasis value.

    :   **Parameters:**: `newDoNotOperateOnPerClassBasis` - The new DoNotOperateOnPerClassBasis value.

---


### minTermFreqTipText

```
public java.lang.String minTermFreqTipText()
```

:   Returns the tip text for this property.

    :   **Returns:**: tip text for this property suitable for displaying in the explorer/experimenter gui

---


### getMinTermFreq

```
public int getMinTermFreq()
```

:   Get the MinTermFreq value.

    :   **Returns:**: the MinTermFreq value.

---


### setMinTermFreq

```
public void setMinTermFreq(int newMinTermFreq)
```

:   Set the MinTermFreq value.

    :   **Parameters:**: `newMinTermFreq` - The new MinTermFreq value.

---


### lowerCaseTokensTipText

```
public java.lang.String lowerCaseTokensTipText()
```

:   Returns the tip text for this property.

    :   **Returns:**: tip text for this property suitable for displaying in the explorer/experimenter gui

---


### getUseStoplist

```
public boolean getUseStoplist()
```

:   Gets whether if the words on the stoplist are to be ignored (The stoplist
    is in weka.core.StopWords).

    :   **Returns:**: true if the words on the stoplist are to be ignored.

---


### setUseStoplist

```
public void setUseStoplist(boolean useStoplist)
```

:   Sets whether if the words that are on a stoplist are to be ignored (The
    stop list is in weka.core.StopWords).

    :   **Parameters:**: `useStoplist` - true if the tokens that are on a stoplist are to be ignored.

---


### useStoplistTipText

```
public java.lang.String useStoplistTipText()
```

:   Returns the tip text for this property.

    :   **Returns:**: tip text for this property suitable for displaying in the explorer/experimenter gui

---


### setStemmer

```
public void setStemmer(Stemmer value)
```

:   the stemming algorithm to use, null means no stemming at all (i.e., the
    NullStemmer is used).

    :   **Parameters:**: `value` - the configured stemming algorithm, or null **See Also:**: `NullStemmer`

---


### getStemmer

```
public Stemmer getStemmer()
```

:   Returns the current stemming algorithm, null if none is used.

    :   **Returns:**: the current stemming algorithm, null if none set

---


### stemmerTipText

```
public java.lang.String stemmerTipText()
```

:   Returns the tip text for this property.

    :   **Returns:**: tip text for this property suitable for displaying in the explorer/experimenter gui

---


### setStopwords

```
public void setStopwords(java.io.File value)
```

:   sets the file containing the stopwords, null or a directory unset the
    stopwords. If the file exists, it automatically turns on the flag to
    use the stoplist.

    :   **Parameters:**: `value` - the file containing the stopwords

---


### getStopwords

```
public java.io.File getStopwords()
```

:   returns the file used for obtaining the stopwords, if the file represents
    a directory then the default ones are used.

    :   **Returns:**: the file containing the stopwords

---


### stopwordsTipText

```
public java.lang.String stopwordsTipText()
```

:   Returns the tip text for this property.

    :   **Returns:**: tip text for this property suitable for displaying in the explorer/experimenter gui

---


### setTokenizer

```
public void setTokenizer(Tokenizer value)
```

:   the tokenizer algorithm to use.

    :   **Parameters:**: `value` - the configured tokenizing algorithm

---


### getTokenizer

```
public Tokenizer getTokenizer()
```

:   Returns the current tokenizer algorithm.

    :   **Returns:**: the current tokenizer algorithm

---


### tokenizerTipText

```
public java.lang.String tokenizerTipText()
```

:   Returns the tip text for this property.

    :   **Returns:**: tip text for this property suitable for displaying in the explorer/experimenter gui

---


### getRevision

```
public java.lang.String getRevision()
```

:   Returns the revision string.

    :   **Specified by:**: `getRevision` in interface `RevisionHandler` **Overrides:**: `getRevision` in class `Filter`
    :   **Returns:**: the revision

---


### main

```
public static void main(java.lang.String[] argv)
```

:   Main method for testing this class.

    :   **Parameters:**: `argv` - should contain arguments to the filter: use -h for help


---


|  |  |  |
| --- | --- | --- |
| **Overview** \| **Package** \| **Class** \| **Tree** \| **Deprecated** \| **Index** \| **Help** \| **Weka's home** | |  |
| **PREV CLASS**   **NEXT CLASS** | **FRAMES**    **NO FRAMES**     **All Classes** |  |
| SUMMARY: NESTED \| FIELD \| CONSTR \| METHOD | DETAIL: FIELD \| CONSTR \| METHOD |  |


---
